# Sensitization and Interoception as Key Neurological Concepts in Osteopathy and Other Manual Medicines

**DOI:** 10.3389/fnins.2016.00100

**Published:** 2016-03-10

**Authors:** Giandomenico D'Alessandro, Francesco Cerritelli, Pietro Cortelli

**Affiliations:** ^1^Clinical-based Human Research Department, Centre for Osteopathic Medicine CollaborationPescara, Italy; ^2^Accademia Italiana Osteopatia TradizionalePescara, Italy; ^3^Department of Neuroscience, Imaging and Clinical Sciences “G. D'Annunzio” University of Chieti-PescaraPescara, Italy; ^4^ITAB-Institute for Advanced Biomedical Technologies, “G. D'Annunzio” University of Chieti-PescaraPescara, Italy; ^5^Department of Biomedical and Neuromotor Sciences, Bellaria Hospital, University of BolognaBologna, Italy; ^6^IRCCS Istituto delle Scienze Neurologiche di Bologna, AUSL di BolognaBologna, Italy

**Keywords:** osteopathic medicine, autonomic nervous system, interoceptive paradigm, allostasis, homeostasis, inflammation, nociception

## Abstract

Historically, approaches used in manual medicine to explain patient reported symptoms have been focused on the so-called exteroceptive paradigm. Arguably, this mindset lacks an appropriate “reading system” able to interpret musculoskeletal disorders from a different perspective, where the properties of the nervous system are embraced into a more holistic and functional-related context. Interestingly, if the underpinning mechanisms of a given treatment scenario/effect are taking into account, the majority of research outcomes focuses on a proprioceptive/exteroceptive explanation, leaving ting aside the additional or even central role of interoception. Currently, to date, the application of theoretical knowledge acquired on the relatively recent neuroscientific concepts and evidence concerning of interoception, sensitization, touch, autonomic functions, inflammation, and pain into a clinical/research manual medicine scenario is lacking, even if theoretically, the impact on the possible etiological mechanisms and treatment effects seems to be important. Here, we propose the conceptual foundations for a new way of interpreting and reading patients' clinical reported outcomes scenario based on interoception and sensitization. We argue that this will provide a foundation to create the ground for future research focusing on the hypotheses that manual therapies, specifically osteopathy, can intercede with sensitization states, at all levels, using interoceptive pathways.

## Introduction: Input—central elaboration—output

Interoception can be described as the moment-to-moment representation process of body sensations coming from the body itself (Craig, 2002). A broader definition considers interoception as a multi-dimensional construct, which includes how people evaluate and react to these sensations (Cameron, 2001). Interestingly, several health problems involve altered interoceptive processes, including chronic pain (Schmidt et al., 1989), post-traumatic stress disorder (Wald and Taylor, 2008), affective disorders (Paulus and Stein, 2010), addiction (Naqvi and Bechara, 2010), eating disorders (Pollatos et al., 2008; Herbert and Pollatos, 2014), somatoform disorders (Mirams et al., 2012; Schaefer et al., 2012), and dissociative disorders (Hankin, 2012; Michal et al., 2014; Sedeno et al., 2014).

Sensitization is defined as the neurologically-based amplification response produced by repeated stimuli. To date, evidence consistently highlights that several subgroup of patients, with or without pain-related syndromes, exhibit a documented sensitization (Table 1). Notwithstanding this, it is still unclear how to recognize a sensitization state using clinical objective measure and tests (see Nijs et al., 2010, for guidelines).

**Table 1:** **List of documented medical conditions with documented sensitization state**.

**Medical condition**	**References**
Neuropathic pain	Baron et al., 2013; Nijs et al., 2014
Chronic pelvic pain	Baron et al., 2013
Irritable bowel syndrome	Verne and Price, 2002
Rheumatoid arthritis	Meeus et al., 2012
Chronic whiplash associated disorders	Curatolo et al., 2001
Endometriosis	Giamberardino et al., 2014
Migraine	Aguggia et al., 2013
Chronic low back pain	Giesecke et al., 2004
Tension-type headache	Ji et al., 2003
Fibromyalgia	Vierck, 2006
Temporomandibular disorders	Maixner et al., 1998
Shoulder pain	Borstad and Woeste, 2015; N Sanchis et al., 2015
Primary, secondary, and widespread hyperalgesia	Bourke et al., 2015
Allodynia	Coppola et al., 2013
Spontaneous pain	Baron et al., 2013
Hypersensitivity to bright light, mechanical pressure	Nijs et al., 2010
Cold temperature	Kasch et al., 2005
Heat temperature	Meeus et al., 2008
Electrical stimuli	Banic et al., 2004
Stress, emotion and mental load	Nijs et al., 2010

The close link between interoception and sensitization (Flor et al., 2004), the individual time-course responses to sensitization (Baron et al., 2013) and the interpersonal clinical variability, create a scenario in which practitioners have to deal with a range of clinical circumstances possibly characterized by the following:

1. *unexplainable* symptoms, i.e., chest pain may depend upon aberrant pain processing from the esophagus due to sensitization of spinal dorsal horn cells and supraspinal centers (Mertz et al., 1998);
2. *indecipherable* pathogenesis, i.e., angina attack could be referred to the site of an old vertebral fracture (Henry and Montuschi, 1978);
3. clinical *heterogeneity*, considering the autonomic effects as relevant co-aspect of patient's clinical manifestations;
4. *causal clinical validity of instrumental exams*, i.e., the disease progression of osteoarthitis seems to be better associated with sensitization than with the actual joint destruction assessed by radiological scorings (Arendt-Nielsen et al., 2010);
5. *unintelligible* treatment outcomes, i.e., referred muscle hyperalgesia could persist even long time after the disappearance of primary focus in the viscous (Giamberardino, 2003);
6. *unforeseeable* prognosis, i.e., after an initial whiplash trauma, the presence of a sensitization process is important to predict the development of chronicity (Sterling et al., 2003);
7. clinical vs. scientific uncertainty of effects and mechanisms of therapies.

The seven above-mentioned points could highlight common heterogeneous scenarios in daily clinical practice, which in turn could impair the practitioners' ability to achieve better health outcomes to their patients. However, the scientific neurological underpinnings, mainly based on interoception and sensitization, found in the last two decades of research, may arguably revolutionize the way in which practitioners could “interact” with their patient in the clinical context.

The aim of the present review is to introduce, discuss and transfer the emerging concepts of interoception and sensitization to the context of manual medicine, specifically osteopathy. To this end, we propose an interdisciplinary, and innovative paradigm (the “interoceptive paradigm”) to interpret patients' signs and symptoms as well as patient's phenomenological changes and possibly use it for further clinical- and lab- based research.

## The afferent system: The input stimuli to the CNS

The modern classification of the sensory system evolved from the work of Sir Charles Sherrington who codified the senses into teloreceptive (vision and hearing), proprioceptive (limb position), exteroceptive (touch, including temperature and pain), chemoreceptive (smell and taste), and interoceptive (visceral) modalities (Sherrington, 1906). However, in the light of recent findings on neurofunctional anatomy, the sensory system can be divided into: teloreceptive, exteroceptive/proprioceptive (also classified as A/sensory system), and interoceptive/nociceptive (otherwise named B/sensory system; Craig, 2002). The differences between A and B system arealso embryologically proven: the development of small-diameter interoceptive afferents originating from small (B) cells is coordinated with the development of lamina 1 cells and represents a well distinct entity from the large-diameter exteroceptive afferents originating from large (A) cells that project to the deep dorsal horn, not connecting with lamina I neurons (Prechtl and Powley, 1990; Woodbury et al., 2001). This embryological difference implies not only different anatomical compounds (i.e., type and distribution of receptors, type of primary afferent nerve fibers, type of central afferent pathways), but also unique functional and physiological features (i.e., label lines, type of sensation—epicritic vs. protopathic, fiber threshold, habituation process, distinct phycophysically feelings). These characteristics generate two clear and distinct roles in decoding external and internal stimuli.

The stimuli can be diverse and organized by type—metabolic, physics, chemical, mechanical, and fluidic—upon the function of time—acute or chronic—frequency and intensity—low vs. high. Therefore, the ability of the two sensory pathways to detect variations (the major characteristics of any receptors) producing action potentials, initiates the process of selectively decoding stimuli coming from the outside and from the inside. Consequently, animals have the ability to decode, process, perceive the external (mainly using the teloreceptive and exteroceptive that is the A/sensory system) and the internal milieu (typically utilizing the interoceptive B/sensory system).

## Interoception

Interoception has been recently reinterpreted by Craig as “the sense of the physiological condition of the entire body” (Craig, 2002), not merely the input coming from the viscera as historically described by Sherrington. This system is an ongoing homeostatic afferent pathway, argued as the sensory complement of the ANS (Craig, 2013), that carries signals from small-diameter Adelta and C primary afferent fibers that represent the physiological status of every tissue of the body. Once homeostatic information from tissues are decoded, then they are conveyed up to the anterior insula after making synaptic relays at different levels (spinal cord–lamina I and II, brainstem–homeostatic regions, thalamus). At the insular level, a meta-representation of the perception of self-emerged as a feeling (sentient) entity, which is a pre-stage for emotional awareness. Detailed reviews of the interoceptive evidence are available elsewhere (Craig, 2002, 2003, 2009).

Converging neurobiological evidence pointed out that the insular cortex (IC) is a critical hub for multimodal interoceptive integration. Thus, the IC has been implicated in interoceptive processes, such as awareness of bodily sensations (Khalsa et al., 2009), but also exteroceptive processes, such as perception of pain (Brooks et al., 2002; Gramsch et al., 2014), taste (Gagnon et al., 2014; Iannilli et al., 2014; Parabucki and Netser, 2014; van den Bosch et al., 2014), smell (Kurth et al., 2010), and touch (McGlone et al., 2014). Furthermore, emotional domains overlap in the anterior insula together with the interoceptive and exteroceptive scenario (Kurth et al., 2010), suggesting an underlying commonality (Critchley et al., 2002). As matter of fact, the insula has been proposed a convergence point between internal and external milieus (Azanon and Soto-Faraco, 2008; Mazzola et al., 2009; Azanon et al., 2010; Ibanez and Manes, 2012). In addition, it has been showed that external signals might also be considered as body-mapped signals of an interoceptive peripersonal space (Couto et al., 2015), particularly in the context of pain where afferent signals could be conceived as an extension of interoceptive processing to peripersonal space (Ferri et al., 2013). Notably, based on new evidence emerging from the field of touch in relation to interoception, it can be proposed the existence of an “interoceptive touch” (also referred as gentle/affective touch), which is mediated by low mechanical threshold C fibers (named C-tactile fibers or CTs), and whose analyses take place in the interoceptive stations, that is, lamina II of the spinal cord, thalamus, insular cortex (McGlone et al., 2014).

Taken together, available evidence indicates that sensory information (like nociception and touch) may be integrated by insular networks in a peripersonal-like fashion way and then further processed by emotional awareness and social behavior mechanisms.

## Central elaboration: The sensitization state

Sensitization is generally defined as a non-associative learning process in which repeated stimuli bring to a progressive amplification of a response (Ursin, 2014). Sensitization has been considered a form of “nociceptive” memory because of similarity between its mechanisms with memory mechanisms (Ji et al., 2003). Considering the neuroanatomical and neurophysiological compound, it is possible to distinguish peripheral sensitization (PS) from central sensitization (CS). PS is defined as an increased responsiveness and, therefore, reduced threshold of nociceptors to stimulation (Sandkuhler, 2009). It has been argued that it has an protective role (Nijs et al., 2014) as increasing pain sensitivity into the site of inflammation (Ji et al., 2003) can prevent further damages (Sandkuhler, 2007). Indeed, PS is characteristic of tissue where inflammatory mediators such as prostaglandin E2, bradikinin, nerve growth factor, substance P (SP) are released altering, in turn, threshold and kinetics of receptors and ion channels of the nociceptive Adelta and C fiber nerve endings (receptor' sensitization). PS is clinically expressed through primary hyperalgesia (increased pain sensitivity at site of injury; Cervero, 2009; Sandkuhler, 2009) and allodynia (pain in response to a non-nociceptive stimulus; Sandkuhler, 2009). CS is a cellular process of increased excitability (Sandkuhler, 2007) that occurs within the CNS. CS includes altered sensory processing in the CNS, such as: (1) alterations of the descending inhibitory pathways arising from the periacqueductal gray matter and the rostral ventral medulla (Meeus et al., 2008), (2) temporal summation of second pain (wind-up; Arendt-Nielsen et al., 1994).

Historically, along the CNS, the spinal cord is the first station in which CS was found. Spinal cord sensitization is characterized by: (1) reduced threshold, (2) increased receptive field sizes, and (3) greater evoked responses in hyperexcitable spinal neurons of dorsal horn as a result of a short barrage of nociceptor input (Woolf and Wiesenfeld-Hallin, 1986; Woolf, 1993). The phenomenon is known as “activity-dependent CS” (Ji et al., 2003) or “homosynaptic facilitation,” and is characterized by the release of several neurotransmitters including SP (see Ji et al., 2003; Sandkuhler, 2009 for reviews). Spinal cord sensitization is a multifaceted phenomenon that has at least three, albeit secondary, actors: (1) A-afferent system, (2) spinal glial cells, and (3) ventral horn motoneurons. (1) Following both peripheral inflammation and nerve injury, it has been showed a phenotypic switch in some large A-system (non-nociceptive), DRG neurons that begin to express key molecules typical of CS, that is SP and BDNF (Neumann et al., 1996; Mannion et al., 1999). (2) Spinal glial cells have an intermediary role between the initial insult and neuronal plastic changes leading to pain amplification (Sandkuhler, 2009), and microglia in dorsal horn seems to have a particular role in inducing neuropathic pain (Watkins et al., 2001). Notwithstanding this, after a peripheral injury or inflammation, microglia (Aldskogius and Kozlova, 1998), and astrocytes (Lee et al., 2009) in spinal dorsal horn rise in number. (3) Although research on CS only indirectly focuses on ventral horn motoneurons by using the withdrawal reflex—a surrogate of enhanced nociception (Sandkuhler, 2009)—, recently it has been described a direct bradykinin-mediated sensitization of spinal lumbar motoneurons of rats (Bouhadfane et al., 2015).

Although, the precise mechanisms remain still understudied, it appears that a mixed of events is necessary to start and sustain the sensitization state in the spinal cord, clinically mainly showed with secondary hyperalgesia, that is an increased pain sensitivity in an area adjacent to the site of injury (Sandkuhler, 2009).

Several research studies have been conducted to examine neuronal sensitization at higher levels along the interoceptive pathway, in particular: (a) spinothalamic tract (Simone et al., 1991; Willis, 2002); (b) brainstem: rostroventral medulla (Porreca et al., 2002); and trigeminal nuclei (Hu et al., 1992)—especially trigeminal subnucleus caudalis (Cao et al., 2013; Wang et al., 2013); (c) diencephalum, thalamic neurons (Park et al., 2006; Kaneko et al., 2011), in the thalamic-anterior cingulate pathway (Shyu and Vogt, 2009), hypothalamic neurons (Peng et al., 2011; Daviu et al., 2014; Donnerer and Liebmann, 2015), in the hypothalamic-pituitary-adrenal axis (Daviu et al., 2014); (d) telencephalic level, including the anterior cingulate cortex (Wei and Zhuo, 2001), amygdala (Neugebauer and Li, 2003), and insular cortex (Qiu et al., 2014).

Collectively, the studies on sensitization begin to show that the PS is a well-studied phenomenon with clearly identified biological pathways. On the contrary, a part from spinal cord sensitization, CS is still an on-going area for research, which showed several characteristics according to the neuronal level considered, but a unified central sensitization state scenario is still lacking.

## The efferent system: The vegetative-somatic dichotomy

The efferent pathway might be divided into a “somatic” and “vegetative” systems throughout the central (upper neuron) and peripheral (lower neuron) nervous system. The “somatic” system is composed by all those tracts which control the motor movements, the “vegetative” pathway generates control on all the functions out of the control of the conscious self.

### Vegetative output

John N. Langley coined the terms “autonomic nervous system” and “parasympathetic nervous system” about the turn of the twentieth century (Langley, 1921), to describe a system that is autonomous, involuntary, and regulates the body's “inner world.” However, the classical distinction between sympathetic and parasympathetic has been recently reviewed in the light of differential responses to stressors and differential involvement in pathophysiological states (Buijs et al., 2003; Buijs, 2013) between the various parts of the autonomic nervous system (ANS). Goldstein proposed that the ANS has at least five components with specific functions: the sympathetic noradrenergic system, the sympathetic cholinergic system, the parasympathetic cholinergic system, the sympathetic adrenergic system, and the enteric nervous system (see Goldstein, 2001, 2006, 2013b; Goldstein and McEwen, 2002, for reviews). In addition, the ANS should not only be seen as a system merely carrying out the commands of the brain; it also functions as a reflex circuit, using the sensory feedback of the organs, to change and precisely adapt the its output in order to adjust the physiological state of the body.

Although much is known about the organization of the ANS output to organs, there is relatively little knowledge regarding the feedback of organs to the brain. It is safe to assume that every organ has the capacity to reach the brain, i.e., via the release of hormones, and thus to provide feedback to the control center of the ANS, mainly using the interoceptive pathway. Most of these metabolic signals may be aimed at regulating the function of the organ in a “reflex” manner, but there is also evidence that this feedback may influence the function of other organs, or behavior, via neuronal sensory feedback (Uno et al., 2006; Warne et al., 2007). This mechanism has been also described in the context of inflammation as neurogenic inflammatory mechanism or more recently “neurogenic neuroinflammation” (Xanthos and Sandkühler, 2014) to define the participation of afferent nerve fibers (mainly amyelinated afferent C fibers–B/afferent system), using an antidromic transmission, to local inflammatory reaction in response to local metabolic modifications due, for example, to infection, trauma, stress, hormonal changes, thus variations in the interoceptive milieu. The aim is to maintain the integrity of the conditions of life within the internal environment,—originally the “milieu intérieur” (Bernard, 1912) then extended to “homeostasis” (Cannon, 1929)—through a mechanism of allostatic adaptation (McEwen, 2007), which can involve the release of key substances like SP, glucocorticoids, catecholamines, and different cytokines (McEwen, 2007; Goldstein and Kopin, 2008).

The active process of responding to challenges is called “allostasis.” This involves several mediators, including autonomic, cortisol, immune/inflammatory, metabolic, and neuromodulators within the brain, that interact non-linearly and promote tuning adaptation in the short term. Overuse (i.e., too much stress) or dysregulation among the mediators (e.g., too much or too little cortisol; too much or too little inflammatory cytokines) can produce cumulative changes that is referred to as “allostatic load and overload” (McEwen, 1998), which in turn can produce sensitization state. This allostatic load, a wear and tear response, produced by the repeated activation of adaptive mechanisms, can last for long and eventually result in a significant alteration of physiological resilience systems (McEwen et al., 2015a,b), which can produce exacerbation of clinical symptoms, including chronic pain.

In addition, several studies demonstrated as the neurogenic inflammation may be evident also at a distant site from the original exposure (Black, 2002). This can be established through the use of different but specific metabolites (i.e., SP), mechanisms (i.e., axon reflex, reverberation), and systems (i.e., immune system; see Xanthos and Sandkühler, 2014, for a review). Furthermore, recent available evidence suggests that neuronal activity in primary afferent peptide C nerve fibers or higher-order neurons is sufficient to elicit neurons in the spinal cord, vascular cells and innate and adaptive immune cells (Xanthos and Sandkühler, 2014).

Interestingly, the central control of the outflow of sympathetic nervous information is strategically organized as a long chain of motor neurons in the intermediolateral column of the spinal cord. This segmental organization allows the ACh-producing motor neurons to establish synapses with different and multiple ganglions along the spinal cord which contain neurons that use different neurotransmitters (i.e., noradrenaline/norepinephrine, neuropeptide Y; Lundberg et al., 1983). This anatomical and functional scenario permits to widen the vegetative output responses to different organs and tissues, creating the neurological ground for modifying the functions of distant bodily sites.

### Muscle-skeletal output (alfa gamma precision and strength)

The second output pathway is the somatic efferent that is characterized by a central and peripheral component. The former is based on a series of tracts, which transport different information from different brain areas (i.e., motor cortex, cerebellum, basal ganglia, forebrain, midbrain) addressing different functions. The latter is characterized by specific efferent somatic neurons (alfa, beta, and gamma motorneurons) interconnected in a fashion-like function (i.e., alfa-gamma coactivation mechanism) controlling the striate muscles of the all body for precision and strength (cortico-spinal tract from the motor cortex), adjustment of head position in response to visual/auditory information (tetto-spinal tract from the superior/inferior colliculi), balance adjustment (cerebellum-spinal tract–cerebellum, rubro-spinal tract–red nucleus, reticulo-spinal tract–reticular formation).

### Integration between the two systems

The interconnection between the two systems is clear both from a neurological and metabolic perspective. Neurologically, vegetative output, and muscle-skeletal output are centrally integrated and reciprocally modulated in many areas of neural axis. Furthermore, are both integrated with the neuroendocrine system, allowing a high-complex level of integration, crucial to reach a coordinated response to ensure homeostasis (Jänig, 2006). Metabolically, nerves, both somatic and autonomic, are intimately associated with inflammatory cells; this is especially true of mast cells which resemble nerve cells in many respects (Purcell and Atterwill, 1995) and confirmed in the bradykinin-induced plasma extravasation inflammation model (Janig and Green, 2014). Moreover, recent evidence showed a mutual somato-vegetative relationship through the activation of an immune-mediated pathway (Sankowski et al., 2015).

## Clinical applications and implications

“Scientific integrative medicine is not a treatment method or discipline but a way of thinking that applies systems concepts to understand normal physiology and clinical disorders, providing a framework for understanding complex and dynamic challenges to our integrity as organisms and, in turn, for developing novel treatments based on this complexity and dynamism” (Goldstein, 2013b, p. 16).

According to the homeostasis theory, stress is considered a state or a condition, in which expectations mismatch the perceptions of the external or internal environment (Goldstein and McEwen, 2002). This incongruity produces patterned and compensatory responses that can change not only the physiology of a target organ but also the general bodily reaction. Stress can be interpreted in terms of an error signal, due by different sources or triggers (i.e., traumatic injury, psychological condition, genetic and/or acquired diseases), which can reflect the difference between input information as felt, neural central “multimodal” elaboration and a series of effects determined by a regulator, possibly the ANS (Goldstein, 2013a). This concept has an intuitive clinical practice application for interpreting the patient's clinical history and treatment effects.

### Implications in manual therapy, specifically in osteopathy

As general rule, the above-mentioned concepts can be applied at any (para-) medical approach including those methods that use a touch-based practice. For the purpose of this review, the following section will focus more on the translation of sensitization and interoception concepts into the field of osteopathy, a drug-free manual medicine approach, which uses touch and manipulation as procedures to diagnose, evaluate and treat (Cerritelli et al., 2015a,b). Osteopathic procedures include a structural evaluation followed by a treatment. The structural evaluation aims to diagnose somatic dysfunctions. It includes an accurate manual assessment of the skull, spine, pelvis, abdomen, upper, and lower limbs to locate bodily areas with an alteration of specific tissue parameters. The treatment includes the application of a range of manipulative techniques aimed at relieving the somatic dysfunctions. Notwithstanding the osteopathic interest, one of the goals of the current review is to propose a modern clinical neuroscience-based praxis to “read and interpret” patients' signs and symptoms, which can be widely shared across disciplines.

Very little research explored the effect of osteopathic manipulation on brain functions. Fryer et al. pointed out that the application of a single high-velocity low-amplitude lumbosacral joint osteopathic manipulation decreases the corticospinal and spinal reflex excitability measured with TMS and EMG suggesting an inhibitory effect at the level of the spinal cord (Fryer and Pearce, 2012).

Moreover, OMT seems to be associated with a reduction of pro-inflammatory substances both *in vitro* (Meltzer and Standley, 2007) and *in vivo* (Licciardone et al., 2012, 2013) hypothesizing an anti-inflammatory role of OMT, although only partially confirmed by recent clinical-based research (Degenhardt et al., 2014).

Osteopathic manipulations, therefore, could reduce the release of cytokines and the sympathetic activity creating a cascade of biological and neurological events that modulate the inflammatory and ANS mechanisms. The application of OMT was demonstrated to influence the ANS, producing a parasympathetic effect (Henley et al., 2008; Giles et al., 2013; Ruffini et al., 2015) and leading, therefore, toward a trophotropic tuning state (Ruffini et al., 2015). Remarkably, no differences were found in sham light-touch control in which only a simple touch was used. This might imply that a “technical” touch must be administered to produce effects. Thus, the operator and its education have a central role.

More recently, starting lab-based evidence showed the effect of specific osteopathic techniques on the enhancement of the lymphatic and immune system (Schander et al., 2012, 2013) by improving the leukocytes count and interleukin-8 (IL-8). Findings were confirmed by a recent 2014 paper where significant differences were detected in the levels of immune molecules, including IL-8, between OMT and sham light-touch control (Walkowski et al., 2014). OMT, therefore, could also have an effect on the immunological profile of specific circulating cytokines and leukocytes. As suggested by Xanthos and Sandkühler, treatments and interventions that are targeted at various levels to inhibit the source of inflammation and neuroinflammatory processes, or to promote the resolution of inflammation, would be recommended to interrupt the neurogenic neuroinflammatory vicious cycle (Xanthos and Sandkühler, 2014). Hypothesising that the osteopathic treatment would fulfill those requirements, in particular the anti-inflammatory action, it could be argued that being exposed to osteopathy can terminate neuroinflammation and reduce pathological outcomes.

However, although starting evidence tried to explore how osteopathy might work, there is no consensus regarding which “channels” osteopathy uses to produce its effects. In fact, the diagnostic knowledge of manual medicine historically is based and built on the prevalently exteroceptive consideration of symptom (i.e., postural interpretation, muscular chains) through the pure “muscle-skeletal paradigm” or “exteroceptive paradigm” in which (1) proprioceptive/exteroceptive afferent activity is integrated in (2) the central motor systems and (3) the output goes out through the Sherrington's final common pathway (alfa-gamma motor neurons of anterior horn of spinal cord). This kind of “exteroceptive paradigm” has been used, for example, in Korr's hypothesis of hyperactive monosynaptic stretch reflex as explanation for the reduction of range of motion (ROM) (Korr, 1975; Howell et al., 2006). However, considering the routinely clinical practice, it is important for clinicians to recognize that feelings from the body, such as pain, are neurologically distinct from tactile mechanoreception and proprioception at all levels. In fact, osteopathic medicine (OM) practitioners face daily clinical cases that cannot be fully explained by the “exteroceptive paradigm” as it lacks of a “clinical reading system” which is able to consider the patient as a whole and not merely a muscle-skeletal body entity. Thus, a wider approach, able to possibly better explain, evaluate, link and predict patients' signs and symptoms is recommended.

**Figure 1:**
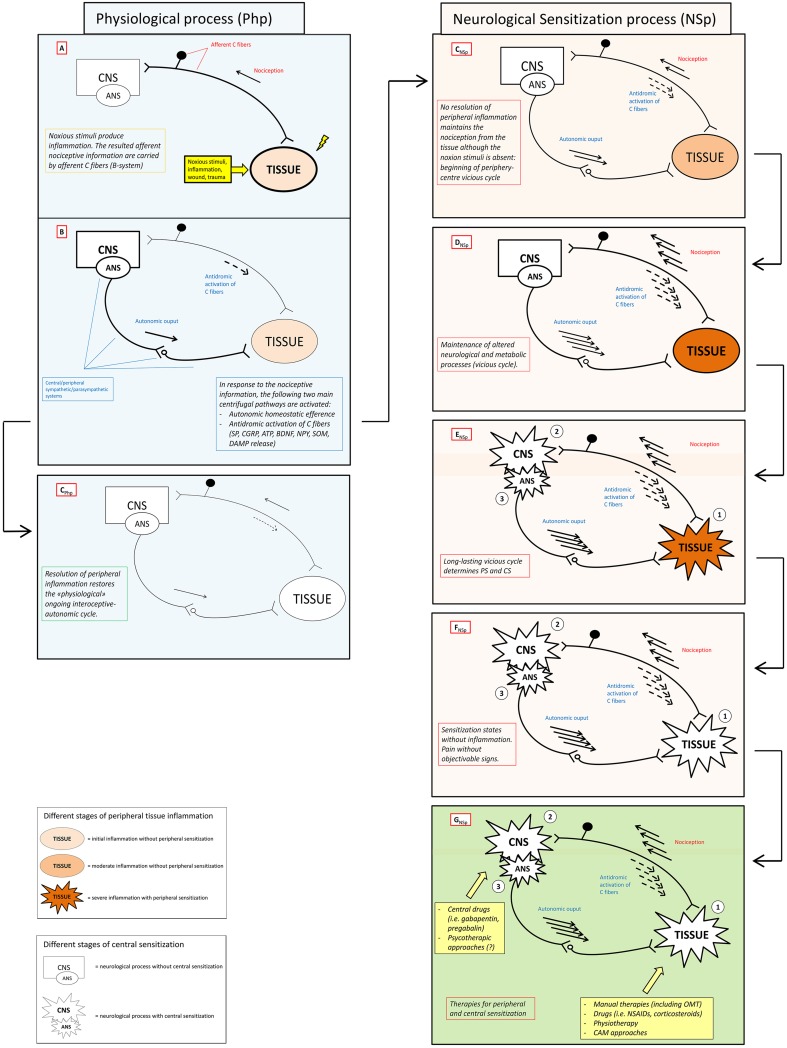
**Neuronal activity in physiological and sensitization processes**. The figure schematically shows the antithetic processes and relative outcomes occurring in physiological (Php) and neurological sensitization process (NSp) after that a stimulus (noxious stimuli, inflammation, wound, trauma) activates the nociceptive afference (box A). Physiologically, antidromic activation of C fibers, the so-called neuorogenic inflammation, and specific autonomic efferences (box B) sustain peripheral healing process restoring both homeostasis and physiological ongoing interoceptive-centrifugal communication between periphery and CNS (box C_Php_). In the right column (pink boxes) lacking of resolution of peripheral inflammation sustains nociceptive afference with a consecutive amplification of centrifugal phenomena (boxes C_NSp_ – D_NSp)_ that can become maladaptive or neurotoxic, see Xanthos and Sandkuhler for details (Xanthos and Sandkühler, 2014). Maintenance of this metabolic-neurological TISSUE-CNS vicious cycle (box E_NSp_)could bring to PS (1) and CS (2) as well as to a never-ending self-maintenance inflammation state (3) (box F_NSp_). Several therapies (box F_NSp)_, including OMT, could be theoretically administered to solve neurological sensitization in different clinical conditions where PS and/or CS are present. CNS, central nervous system; ANS, autonomic nervous system; PS, peripheral sensitization; CS central sensitization; SP, substance P; CGRP, calcitonin gene-related peptide; ATP, adenosine triphosphate; BDNF, brain-derived neurotrophic factor; NPY, neuropeptide Y; SOM, somatostatin; CB, cannabinoid; DAMP, danger associated molecular patterns; OMT, osteopathic manipulative treatment; NSAIDs, non-steoridal anti-inflammatory drugs; CAM, complementary alternative medicine.

Here we propose the “interoceptive paradigm” in which (1) altered (acutely and chronically) interoceptive information lead to (2) neurological “sensitization states” (SS) that express their dysfunction through (3) an altered firing of the autonomic nervous system (ANS), which in turn (4) brings the peripheral tissue to an hypersensitivity state and, thus, creating the ground for a (5) vicious metabolic and neurologic cycle (positive feedback loop) and rapid system failure (Figure 1). The recognition of this paradigm will impact the clinical practice with several advantages/benefits:

- Appropriate clinical interpretation of symptoms with respect to the causal and pathogenetic aspects;
- Pertinent ability to “read” and “elucidate” the clinical history, linking aspects related to organs functions, neurology, and pathophysiological adaptation/compensation;
- Adequate comprehension of roles in the mutual doctor-patient relationship.

Although, according to some authors (Sandkuhler, 2007; Cervero, 2009), sensitization is a status of increased excitability at the cellular level, it can be also used in a broader perspective, at clinical and behavioral levels (Coppola et al., 2013; Ursin, 2014), to describe an increased sensitivity to pain or an increased excitability of the central nervous system (CNS). It has been shown that an input is necessary to start, sustain, or impair the sensitization process (Melzack et al., 2001; Affaitati et al., 2011; Baron et al., 2013). Being stimulus-dependent, sensitization can be seen has an adapting response of CNS to environmental challenges presenting through the nociceptive afference that not necessarily has to become subjectively perceived (Treede et al., 1999; Kidd and Urban, 2001; Sandkuhler, 2009). This is remarkable from a case history taking and, more generally, a diagnostic point of view. From a therapeutic perspective, it is important to consider that touch could be a potential input able to modify the sensitization state. Indeed, emerging evidence showed the relevance of a gentle/affective touch to elicit CT fibers and therefore modulate the interoceptive pathway. This produces a central reaction which in turn evokes a series of neurological events that brings the ANS to respond to a given stimulus. This type of touch is different from the well-known “exteroceptive touch” that is mediated by the low-threshold mechanoreceptors (LTMs) innervated by Abeta afferents. This “exteroceptive touch” is able to rapidly detect, discriminate and identify external stimuli to prepare an appropriate sensorimotor transformation. On the contrary, CTs, found only in hairy but not glabrous skin, respond to slow (1–10 cm/s), weak (0.3–2.5 mN), mechanical stimuli (Perini et al., 2015). It has been shown that CTs are also temperature tuned at 32°C and are associated to sensual touch (Ackerley et al., 2014; Perini et al., 2015) suggesting not only a socially-relevant function (Perini et al., 2015) but also a possible role in the neurodevelopment during the perinatal period (Bystrova et al., 2009).

Translating this evidence into a clinical touch-based manual medicine perspective, although the role of CTs in pain modulation (especially allodynic experience) remains an open question (Nagi et al., 2011; Delfini et al., 2013), the interoceptive affinity of a well determined type of touch able to activate CTs provide a rational basis for complementary medical approaches like therapeutic touch (Craig, 2013). In addition, these findings reveal that feelings from the body, such as pain, are inherently linked with autonomic conditions, such as plasma extravasation or cardiac rhythmicity, because they are, respectively, sensory and motor aspects of the same homeostatic system. Moreover it is important to consider that also the exteroceptive touch mediated by low-threshold-mechanoceptors could modulate efferent activity of the ANS, especially locally (Jänig, 2006; Craig, 2014). Thus, the relationship between manual therapies effects, specifically osteopathy, and interoception implications can be argued at the light of the prevailing neuroscience literature, although not formally tested.

Continuing to translate neuroscientific paradigms into osteopathy, interestingly, Livingstone (1943) proposed that the afferent activity produced by injured peripheral nerves elicits an abnormal firing pattern within the spinal cord. The author argued that a disturbance occurs in an internuncial pool of dorsal horn interneurons resulting in reverberatory activity that spreads to various areas of the spinal cord, including the sympathetic chain. Increased activity in sympathetic output would interfere with vasoregulation and induce further hypersensitivity of peripheral tissue, leading to increased afferent input and a vicious circle of peripheral-central activity. If this process lasts for long, then a sensitization state is produced, as described above. Therefore, if the osteopathic touch is supposed to produce an anti-inflammatory and hyper-parasympathetic effect, it can be argued that, potentially, modulating the vegetative firing, it can produce positive feedback effects on the sensitization state.

As final remarks, it is paradoxical the difference between the presence of interoceptive and sensitization phenomena in osteopathic clinical practice, and the almost absence of these concepts in the philosophical, diagnostic, and therapeutic body of OM. This paradox becomes dramatic considering the neurological “qualitative” nature (intero/nociceptive) of the ultimate symptom faced in medical setting: the pain. As Craig argued, from a therapeutic perspective it is relevant to consider that when patients report their symptomatology they are possibly giving a description of the condition of homeostatic systems (Craig, 2013). However it is uncertain patients' accuracy in describing internal states (Petersen et al., 2015). As a matter of fact, it could be important listening to and reporting the spontaneous patients' feelings during the treatment phase. It might represent a potential online homeostatic/allostatic feedback, which can be used for optimizing the treatment plan. In addition, it is important to consider that patients emotional/psychological status (i.e., anxiety or fear) is recognized as part of the perceptual process. This is particularly relevant when given self-rated symptoms (i.e., pain and dyspnea) are described (Petersen et al., 2015), generating over- or under- estimation of interoceptive/nociceptive reporting.

Furthermore, the importance of the interaction between brain and body in order to maintain homeostasis has been emphasized. This is not just a matter of a top- down- or reflex regulation, it is also a matter of signals from the organs influencing the functioning of the brain. The output of the CNS to control its autonomic output shows an amazing differentiation; not only there are different neurons, which may influence selectively the parasympathetic or sympathetic motor neurons, there are also different neurons that project to different body compartments. Based on all that information, the brain sets the balance of the different parts of the ANS, changing the emphasis of the ANS output depending on the situation. If that balance is disturbed, either by behavior or by disease of the organ/tissue, this may lead to pathology that may affect the functioning of the whole individual. Several research studies, indeed, support the hypothesis that lack of balance in the autonomic output to a single organ may have effects not only on the organ itself but also on the entire body physiology.

## Conclusions

The current review presented the “interoceptive paradigm” as a theoretical framework to explain how patient's signs, symptoms, and clinical history can be mutually related in clinical practice. Moreover, it suggested that touch-based manual practices, in particular osteopathy that seems to produce anti-inflammatory and hyper-parasympathetic effects, can offer an alternative but unique way to modify temporary or permanent sensitization states throughout the interaction with (treatment of) peripheral tissues. This is supposed to produce a biological and neurological cascade of events that change the interoceptive processes, breaking the vicious cycle of an on-going low threshold inflammatory condition. Therefore, this work proposes the conceptual foundations for a new way of interpreting and reading patients' clinical scenario based on up-to-date neuroscientific concepts. This will possibly create the ground for future research focusing on the concrete possibility of manual therapies, specifically osteopathy, to interactively modify the sensitization states, at all levels, using interoceptive pathways.

## Author contributions

GD, FC, PC conceived the idea and drafted the first version of the paper. PC supervised the manuscript and reviewed the paper for intellectual content. All authors approved the final version.

### Conflict of interest statement

The authors declare that the research was conducted in the absence of any commercial or financial relationships that could be construed as a potential conflict of interest.
